# Annotations

**Published:** 1887-12-24

**Authors:** 


### ANNOTATIONS.

**A Doctor and his Hobby.**

SOME persons are never so happy as when they are mounted on a favourite hobby and riding it to death. At a meeting of the "Bread and Food Reform League," held on Saturday, at the Parkes Museum, Dr. Norman Kerr is reported to have said that ordinary bakers' white bread was "a sham, having the power to satisfy hunger without properly contributing to man's sustenance. The flour of which it was composed lacked the vital portion of the grain of wheat. White bread was almost without brain-forming power, and if it were not supplemented by other food we should become a comparatively brainless race. . . . If we were not to be a nation of jellyfish we must give the matter our immediate and close attention." Miss Yates, who is well-known in connexion with the whole-meal bread movement, followed in the same strain. There is nothing more satisfactory to a listener or reader than definiteness in a teacher. But when a man is definite it is highly desirable that he should be definitely right. Dr. Norman Kerr makes the most unqualified and categorical statements. If he is correctly reported, those statements do not admit of a shadow of doubt or denial. But Dr. Norman Kerr is a medical man, and therefore accustomed to look at questions from a purely scientific standpoint. The general public in reading his utterances will take it for granted that they are in accord with the known facts of science bearing on the subject. We would, however, venture to ask him what are his grounds for such wholesale condemnation of one of the staple articles of an Englishman's food? Such statements should not be rested on the shifting foundations of mere opinion, however plausible the opinion may be, and however supported by great names. There are now recognised methods of ascertaining the food value of any given substance. But these methods are not confined to the laboratory. Experiments can be made on a larger, indeed on an universal scale; and we venture to suggest that the results witnessed in the wide field of the world, and manifested over a period of years cannot be set aside on the authority of a chemical experiment, or of the effects of different diets given to a couple of dogs. It is not in this way that progress in science is made.

**Whole-Meal Bread.**

IT is as possible to injure a man or an article of consumption by over-laudation as to destroy it with faint praise. Some people cannot express their satisfaction with their own possessions in any way which will please them so well as by depreciating the similar possessions of their neighbours. It will not content them that all their own geese are swans; they must also insist that all their neighbour's swans are geese. In the same way when some men take up new causes their former enthusiasms sink into comparative insignificance. Whole-meal bread, which is a very wholesome substance, and probably more suitable for the majority of people than bread made of unmixed white flour, seems likely to be discredited by the intemperate advocacy of its friends. Granted that it is highly nutritious, and in every way suited as aliment for the human stomach—more suited, in fact, than bread made of any other substance, it does not therefore follow that other bread stuffs are of no value or positively injurious. A single comparative experiment on a couple of dogs taken alone proves nothing at all. The baker might well answer, "You tell me about your two dogs; I can tell you about two hundred millions, and as many more as you please, of human beings who have had no other bread but that made of white flour from infancy to the end of life, and who have neither been brainless nor turned to jellyfish, but who, on the contrary, have been strong-limbed and keen-witted, and have lived to a ripe old age." If the physiological laboratory is going to make us close our eyes to all the great facts which are transpiring in the larger laboratory of the world through the centuries, the sooner the experiment-room is closed the better. Let us by all means make use of every possible method of increasing our knowledge and rendering it precise and definite. But a weather-glass hung up in a room can never tell us half so convincingly that it rains as a walk in the open air without an umbrella.

**The Influence of Heredity.**

DR. R. W. FELKIN, lecturer on Diseases of the Tropics in the Edinburgh School of Medicine, recently delivered an interesting lecture on this subject to the Edinburgh Health Society. Dr. Felkin is a distinguished African traveller, and has an almost unique knowledge of this particular subject, as illustrated by the native races. He pointed out as a well-known fact that the majority of individuals of the same race are of about the same physical stature. In those cases where both parents possess some well-marked and definite characteristic, a curious result is seen. If, for example, a white man of high intellectual capacity marry a negro woman of great physical beauty and strength, their descendants will be mulattos, who inherit from the father his intellectuality, and from the mother her physical power and beauty. Beggars and criminals, it is well known, are often succeeded by beggars and criminals. The genealogy of the Jukes family in America includes 540 individuals, most of whom are either criminals, paupers, or diseased. Out of thirty-eight persons belonging to one branch of this family, sixteen have been in jail, eleven have led openly disreputable lives, four have been drunkards, the history of three has not been traced, and only four are known to have conducted themselves well. Many similar facts were given which are doubtless more or less familiar to our readers. What we wish to point out is that although heredity exercises a marked influence in certain directions the influence is not such as is incapable of amelioration or modification. For instance, a man may be born with certain inherited physical characteristics which are injurious to him. It is a fact of experience that if he be judiciously managed in infancy, youth, and manhood those characteristics may be very much modified for the better; and other and counterbalancing features may be developed to such a degree as entirely to change his physical or mental or moral character. Marriage is a most important question for those who have unhappy physical or other peculiarities. The marriage of cousins, which is always undesirable, is especially so where there are marked and injurious family characteristics. The subject very properly receives much attention at the hands of certain eminent individuals. It ought, however, to enter into the practical calculations of all intelligent people of both sexes. But though it is eminently desirable that the facts brought to light about heredity should be taken into account, it is not at all a thing to be desired that they should be permitted to dwarf other and equally well-established facts and generalisations based thereupon. There are some people who cannot hold a sound principle without regarding it as the only sound principle in the world. A large generalisation is to them a kind of Moses' rod, whose business it is immediately to swallow up every other rod in existence. They will affirm, for example, that if a man inherits certain peculiarities of mind and character, he must necessarily be the slave of those peculiarities; and that, as a corollary, he cannot be held responsible for any actions which may be the result of his hereditary tendency. That is not only a most dangerous view of the question, but as foolish and unscientific as it is dangerous. The truth is that man is a being capable of almost infinite modification; he can adapt himself to every variety of circumstance. Even the idiot can be so trained and instructed as to perform many of the duties of a capable man. Those who attach too much importance to physical peculiarities and teach the relative insignificance of the mental and moral elements in man may be narrowly, but are certainly not philosophically, scientific. If religious enthusiasts cannot bend the facts of science to their will, neither can narrow scientists, as yet at any rate, explain all the phenomena of mind and morals by any of their mechanical hypotheses. The infinite mystery around and within us is still a mystery in spite both of rushlights and electric lights, and promises to remain so at least during the lifetime of the present generation.

